# Influence of neighborhood-level socioeconomic deprivation and individual socioeconomic position on risk of developing type 2 diabetes in older men: a longitudinal analysis in the British Regional Heart Study cohort

**DOI:** 10.1136/bmjdrc-2023-003559

**Published:** 2023-10-31

**Authors:** Kathryn J Bush, A Olia Papacosta, Lucy T Lennon, Judith Rankin, Peter H Whincup, S Goya Wannamethee, Sheena E Ramsay

**Affiliations:** 1 Population Health Sciences Institute, Newcastle University, Newcastle upon Tyne, UK; 2 Primary Care and Population Health, University College London, London, UK; 3 Population Health Research Institute, St George's University of London, London, UK

**Keywords:** Aging, Diabetes Mellitus, Type 2, Public Health

## Abstract

**Introduction:**

Evidence from longitudinal studies on the influence of neighborhood socioeconomic deprivation in older age on the development of type 2 diabetes mellitus (T2DM) is limited. This study investigates the prospective associations of neighborhood-level deprivation and individual socioeconomic position (SEP) with T2DM incidence in older age.

**Research design and methods:**

The British Regional Heart Study studied 4252 men aged 60–79 years in 1998–2000. Neighborhood-level deprivation was based on the Index of Multiple Deprivation quintiles for participants’ 1998–2000 residential postcode. Individual SEP was defined as social class based on longest-held occupation. A cumulative score of individual socioeconomic factors was derived. Incident T2DM cases were ascertained from primary care records; prevalent cases were excluded. Cox proportional hazard models were used to examine the associations.

**Results:**

Among 3706 men, 368 incident cases of T2DM were observed over 18 years. The age-adjusted T2DM risk increased from the least deprived quintile to the most deprived: HR per quintile increase 1.14 (95% CI 1.06 to 1.23) (p=0.0005). The age-adjusted T2DM HR in social class V (lowest) versus social class I (highest) was 2.45 (95% CI 1.36 to 4.42) (p=0.001). Both associations attenuated but remained significant on adjustment for other deprivation measures, becoming non-significant on adjustment for body mass index and T2DM family history. T2DM risk increased with cumulative individual adverse socioeconomic factors: HR per point increase 1.14 (95% CI 1.05 to 1.24).

**Conclusions:**

Inequalities in T2DM risk persist in later life, both in relation to neighborhood-level and individual-level socioeconomic factors. Underlying modifiable risk factors continue to need to be addressed in deprived older age populations to reduce disease burden.

**What is already known on this topic**

- Previous studies have shown that individuals with the lowest socioeconomic positions and living in the most deprived areas have the greatest risk for type 2 diabetes mellitus (T2DM).
- Few studies have focused on socioeconomic inequalities in T2DM in older age populations and few have investigated the prospective associations of both individual-level and neighborhood-level socioeconomic factors with T2DM incidence.

**What this study adds**

- This study shows that inequalities in T2DM risk in the UK persist into later life, both in relation to neighborhood-level and individual-level socioeconomic factors.

**How this study might affect research, practice or policy**

- Underlying modifiable risk factors continue to need to be addressed in deprived older age populations to reduce the burden of T2DM in the UK.

## Introduction

Around 3.5 million adults in the UK have a diagnosis of type 2 diabetes mellitus (T2DM), and it is estimated that a further 1 million people are living with the condition undiagnosed.^1^ The prevalence of T2DM rises with age. In England, 18% of people aged 65 years or older have T2DM,^2^ and the overall number of older adults affected by T2DM is expected to continue to rise with the UK’s aging population, mirroring global trends.^3^ The burden of T2DM is manifested in the form of acute emergency presentations with hyperglycemia or hypoglycemia, and chronic microvascular and macrovascular complications, such as diabetic neuropathy, retinopathy, nephropathy, and cardiovascular disease (CVD), presenting a complex public health challenge.^4^ However, many of the established risk factors for T2DM, including obesity, diet, smoking, and physical inactivity, are potentially modifiable.^5^ The modifiable risk factors for T2DM are unequally distributed within the UK, with the highest rates of smoking, obesity, and physical inactivity seen within the most deprived populations.^6–8^ These differences, and their subsequent health outcomes, represent “health inequalities,” which the NHS England defines as “unfair and avoidable differences in health across the population….”^9^

Multiple studies have shown that there are socioeconomic inequalities in the distribution and burden of T2DM. Individuals from lower socioeconomic positions (SEPs) are at a greater risk for T2DM.^5^ Studies have also shown that socioeconomic deprivation at the neighborhood level is associated with T2DM and that people living in the most deprived areas are at the highest risk for T2DM compared with those living in less deprived areas.^10–21^ However, despite the increasing proportion of older adults in the UK population and beyond, few studies have focused on socioeconomic inequalities in T2DM in older age populations. To improve population health overall, it is important to understand whether this older age adult subgroup is similar to the previous younger adult subgroups studied, as this will determine whether it is appropriate to continue to direct public health strategy prevention strategies at this population and enable appropriate targeting of initiatives to prevent T2DM in populations at greater risk. The single previous study conducted to assess this association in the UK included only older age women and is therefore only generalizable to approximately half of our population.^11^ We therefore aimed to investigate the relationships between neighborhood-level socioeconomic factors and T2DM incidence over a follow-up period of 18 years in a representative sample of older British men. We also explored the potential roles that individual SEP and known risk factors for T2DM have in explaining the relationship of neighborhood deprivation with T2DM.

## Methods

The British Regional Heart Study (BRHS) is a longitudinal cohort study that was established in 1978–1980 to investigate regional variations in CVD.^22^ A total of 7735 socially representative men aged 40–59 years were recruited from general practices (GPs) from 24 towns across England, Wales, and Scotland. These men underwent physical examination and completed questionnaires at baseline recruitment. The participants have since been followed up via the Office for National Statistics (ONS) (yearly mortality data), postal questionnaires and through two-yearly review of their primary care (or GP) record.

During a 2-year period (1998–2000), all of the surviving participants in the BRHS cohort were invited for a follow-up physical examination and questionnaire assessment. This assessment time point serves as baseline for the analysis in this study. A total of 4252 men aged between 60 and 79 years attended the examination (77% response rate), had fasting blood samples taken, and completed the questionnaires about their health and lifestyle. Follow-up of participants then continued via a biannual review of their GP records and yearly ONS mortality records. This study includes follow-up data collected from 2000 to the end of 2018, allowing all surviving participants 18 years of follow-up.

All participants provided written consent for the study and investigations.

### Incident T2DM cases

Participants were followed up over the time period from 1998–2000 until the end of 2018 for the study outcome of incident T2DM. Prevalent cases of T2DM were excluded at baseline and included men with a diagnosis of T2DM in their medical records, a self-reported diagnosis of T2DM, or a fasting diabetic range glucose (≥7 mmol/L) or hemoglobin A1c (HbA1c) (≥48 mmol/mol) at the baseline examination.^23^ Incident cases were defined as those who had a new clinical diagnosis of T2DM coded in their medical records during the follow-up period.

### Neighborhood-level socioeconomic deprivation

The study exposure “neighborhood-level deprivation” was based on the Index of Multiple Deprivation (IMD) scores at “Lower-layer Super Output Area” (LSOA) for England and Wales, or “Data Zone” for Scotland. LSOAs are produced by the ONS for reporting of small area statistics. They include approximately 1500 residents or 650 households.^24^ Data Zones in Scotland contain an average of 750 people.

Participants’ postcodes of residence in 1998–2000 were mapped to LSOAs or Data Zones to obtain the IMD scores. Deprivation scores were calculated using standardized IMD scores for England (2004),^25^ Scotland (2004),^26^ and Wales (2005).^27^ The IMD scores for England were based on neighborhood measures from 2000 to 2001.

IMD scores are measures of relative socioeconomic deprivation produced by the UK government. The English IMD score is based on seven different domains of deprivation: income deprivation, employment deprivation, education skills and training deprivation, health deprivation and disability, crime, barriers to housing and services, and living environment deprivation.^24^ The Scottish and Welsh IMD scores are similar but not identical to English IMD, which required standardization of IMD scores; the methods used by the BRHS to achieve this have been previously published.^28 29^

### Individual-level SEP

The study exposure of the participants’ *individual-level socioeconomic position (SEP*) was based on their longest-held occupation at study entry (aged 40–59 years). This was used to define participants’ social class according to the Registrar General’s social class classification,^29^ which includes six categories: I (professional occupations), II (managerial and technical occupations), IIIN (skilled occupations: non-manual), IIIM (skilled occupations: manual), IV (semiskilled manual occupations), and V (unskilled manual occupations), with social classes IIIM–V representing the “manual social class” group.

Further information related to individual-level socioeconomic measures was also collected in the 1998–2000 questionnaires to explore participants’ individual-level socioeconomic factors at the study baseline in more depth.^30^ Questions included whether they owned a car, owned their home, the type(s) of pension received, and whether they lived in a home with central heating. Age at leaving full-time education was obtained through an earlier questionnaire in 1996. These measures were combined with social class to obtain a cumulative (or additive) measure of adverse socioeconomic factors across the adult life course, including education, occupation, and living circumstances in older age.

### Covariates

Each participant’s systolic blood pressure (SBP), body mass index (BMI), and serum cholesterol were measured at the 1998–2000 study baseline physical examination using standardized techniques. Information regarding family history of diabetes in a parent or sibling and the T2DM lifestyle risk factors of physical activity levels, smoking status, and alcohol intake was collected from questionnaire data recorded at this study’s baseline. Detailed descriptions of the BRHS 1998–2000 examination process have been previously published.^22 31^

### Statistical analyses

IMD scores were divided into quintiles, with quintile 1 being the least socioeconomically deprived and quintile 5 the most socioeconomically deprived. Cox proportional hazards models were fitted to examine the associations of IMD, social class, and incident diabetes. Proportional hazards assumption was checked and found to hold, particularly for these main variables of socioeconomic factors. HRs with 95% CIs were produced, where the least deprived quintile (quintile 1) and the highest social class (class I) were used as the reference groups for comparison. Models were adjusted first for age (model 1), followed by mutual adjustments for social class or IMD quintile (model 2). The model was further adjusted sequentially for BMI and self-reported family history of T2DM in a parent or sibling (model 3), and then further adjusted for smoking, alcohol, physical activity levels, SBP, and cholesterol levels (model 4).

Similar models were also undertaken to examine the association between a cumulative score for adverse socioeconomic measures and incident diabetes. The cumulative score was based on recruitment and baseline questionnaire data, and participants scored 1 point for each of the following factors: being in a manual occupational social class at study recruitment, not owning a car, not owning their home, receiving only the state pension, no central heating at home, and leaving education at or before 14 years of age. Each individual therefore received a score between 0 and 6, with a score of 6/6 representing a person who is more likely to have been exposed to multiple adverse social risk factors throughout the life course. All elements contributed equal weighting to the score and similar scores have been used previously in studies of diabetes risk.^32^

For the adjustments in the Cox regression models, age, SBP, BMI, and cholesterol were fitted as continuous variables. Other variables in the models included social class according to six levels (I, II, IIIN, IIIM, IV, and V), activity with six levels (inactive, occasional, light, moderate, moderate-vigorous, vigorous), smoking with four levels (never, long-term ex-smoker (>15 years), recent ex-smoker (<15 years), current smoker), and alcohol with five levels (none, occasional (<1 drink/week), light (1–15 drinks/week), moderate (16–42 drinks/week), and heavy (>42 drinks/week)). All analyses were performed in SAS V.9.4.

## Results

A total of 4252 participants (77% of the surviving participants) attended the physical examination and completed the questionnaires in 1998–2000. Of these, a total of 3706 participants without prevalent diabetes were followed up for a period of 18 years; among these participants, 368 new cases (10%) of T2DM occurred during this period. Table 1 presents the baseline characteristics of this cohort according to the IMD quintile of neighborhood-level deprivation. The table shows a graded increase in the proportion of participants from IMD quintiles 1 to 5 for the major modifiable lifestyle risk factors for diabetes, including, obesity, smoking, and physical activity levels, each with a p value for trend <0.0001; the highest levels of these risk factors were observed in the most deprived quintiles.

**Table 1:** Baseline characteristics according to quintile of neighborhood deprivation in the British Regional Heart Study cohort aged 60–79 years

**Quintile of neighborhood deprivation**	**Quintile 1 (least deprived)** **n=852**	**Quintile 2** **n=877**	**Quintile 3** **n=730**	**Quintile 4** **n=632**	**Quintile 5 (most deprived)** **n=609**	**P value for trend**
Sociodemographic data						
Mean age (SD)	68.48 (5.47)	68.26 (5.53)	68.80 (5.68)	68.79 (5.69)	69.09 (5.30)	0.0073
Manual occupational social class	241 (29)	346 (41)	386 (55)	433 (70)	473 (79)	<0.0001
Left education ≤14 years	179 (23)	242 (30)	255 (40)	241 (44)	243 (50)	<0.0001
Do not own a car	40 (5)	80 (9)	114 (16)	147 (23)	206 (35)	<0.0001
Do not own their house	25 (3)	53 (6)	78 (11)	122 (20)	173 (29)	<0.0001
Receive only state pension	53 (7)	107 (13)	125 (19)	138 (24)	169 (32)	<0.0001
No central heating at home	24 (3)	37 (4)	47 (7)	61 (10)	71 (12)	<0.0001
Individual cumulative adverse socioeconomic factors score						
≥3 (high score for adverse socioeconomic factors)	42 (5)	79 (9)	141 (19)	182 (29)	238 (39)	<0.0001
Behavioral risk factors						
Current and recent ex-smokers	139 (16)	196 (22)	177 (24)	196 (31)	241 (40)	<0.0001
Body mass index >30 kg/m^2^	100 (12)	131 (15)	104 (14)	128 (20)	177 (19)	<0.0001
Alcohol intake (moderate and heavy) >16 units/week	138 (16)	163 (19)	126 (18)	115 (19)	139 (24)	0.0047
Inactive (combining inactive, occasional, and light)	358 (44)	399 (47)	389 (55)	357 (58)	354 (61)	<0.0001
Family history						
Family history of ≥1 parents/sibling with diabetes	69 (8)	95 (11)	61 (8)	67 (11)	43 (7)	0.5493
Biological risk factors						
“Pre-diabetic”-range glucose or HbA1c*	156 (18)	153 (17)	123 (17)	124 (20)	115 (19)	0.5097
Hypertensive	600 (70)	628 (72)	517 (71)	476 (75)	437 (72)	0.2171
Raised total cholesterol (>5 mmol/L)	692 (85)	685 (83)	571 (82)	457 (78)	467 (80)	0.0016
Fasting blood biomarkers, mean (SD)						
Glucose, mmol/L	5.61 (0.51)	5.56 (0.52)	5.56 (0.55)	5.57 (0.54)	5.58 (0.56)	0.1646
HbA1c, nmol/mol	4.75 (0.53)	4.83 (0.56)	4.81 (0.53)	4.88 (0.55)	4.84 (0.54)	0.0002
Insulin, mIU/L	9.10 (6.63)	9.22 (6.08)	9.44 (6.69)	9.80 (6.90)	10.35 (14.70)	0.0220
Gamma-glutamyl transferase, U/L	33.35 (47.21)	33.78 (32.30)	33.58 (32.00)	34.98 (47.28)	37.28 (46.04)	0.0651
C reactive protein, mg/L	2.56 (5.00)	3.18 (6.89)	3.48 (6.89)	4.12 (8.12)	3.99 (6.56)	<0.0001
Interleukin 6, pg/mL	2.76 (2.69)	2.90 (2.63)	3.06 (2.80)	3.50 (3.26)	3.75 (3.33)	<0.0001

Data presented as n (%), apart from age and biochemical markers.

% is the proportion of the quintile data available for displaying the given characteristic.

*Fasting plasma glucose level of 5.5–6.9 mmol/L or an HbA1c level of 42–47 mmol/mol (6.0%–6.4%).

HbA1c, hemoglobin A1c.

Table 2 displays the HR with 95% CI for incident diabetes, where the “relative hazard” or risk of diabetes in IMD quintiles 2–5 is compared with the least deprived IMD quintile 1. The age-adjusted risk of incident diabetes showed a graded increase from IMD quintiles 1 to 5; the HR per IMD quintile increase was 1.14 (95% CI 1.06 to 1.23) (p for trend=0.0005). When further adjustment was made for individual social class, the HR estimates weakened, although the trend across IMD quintiles remained statistically significant. The model was further adjusted for BMI and family history of diabetes in a first-degree relative, which weakened the HR estimates further, with the effects of adjustments almost entirely due to BMI. Further adjustment for behavioral and biological risk factors for diabetes (smoking, alcohol intake, physical activity levels, SBP, and cholesterol levels) attenuated the HR in IMD quintile 4 slightly and this was no longer statistically significant. Model 4 in table 2 presents these fully adjusted HRs and CIs after adjusting for smoking, alcohol consumption, physical activity levels, SBP, and cholesterol. Further adjustment for these factors did not materially change the HRs. The full models for the analyses in tables 2–4 are presented in the online supplemental appendix.

10.1136/bmjdrc-2023-003559.supp1 Supplementary data

**Table 2:** HR for incident type 2 diabetes in the BRHS cohort according to IMD quintiles of neighborhood-level deprivation scores

		HR (95% CI)			
	**Cases of incident diabetes**	**Model 1**	**Model 2**	**Model 3**	**Model 4**
Neighborhood deprivation					
IMD quintile 1 (least deprived)	73	1.00	1.00	1.00	1.00
IMD quintile 2	79	1.07 (0.78 to 1.47)	0.97 (0.70 to 1.34)	0.95 (0.69 to 1.32)	0.90 (0.63 to 1.28)
IMD quintile 3	74	1.33 (0.96 to 1.84)	1.15 (0.82 to 1.61)	1.13 (0.81 to 1.59)	1.19 (0.83 to 1.71)
IMD quintile 4	80	1.80 (1.31 to 2.47)	1.52 (1.09 to 2.13)	1.42 (1.01 to 1.99)	1.31 (0.90 to 1.90)
IMD quintile 5 (most deprived)	62	1.46 (1.04 to 2.05)	1.21 (0.84 to 1.73)	1.16 (0.81 to 1.67)	1.16 (0.78 to 1.73)
P value for trend		0.0005	0.0381	0.0865	0.1196
HR per increase in quintile		1.14 (1.06 to 1.23)	1.09 (1.01 to 1.18)	1.07 (0.99 to 1.16)	1.07 (0.98 to 1.17)

Model 1: adjusted for age.

Model 2: further adjusted for individual social class.

Model 3: further adjusted for BMI and family history of diabetes.

Model 4: further adjusted for smoking, alcohol, activity level, SBP, and cholesterol.

BMI, body mass index; BRHS, British Regional Heart Study; IMD, Index of Multiple Deprivation; SBP, systolic blood pressure.

**Table 3:** HR for incident type 2 diabetes in the BRHS cohort according to socioeconomic position (occupational social class)

		HR (95% CI)			
	**Cases of incident diabetes**	**Model 1**	**Model 2**	**Model 3**	**Model 4**
Socioeconomic position					
Social class I (least deprived), n=354	29	1.00	1.00	1.00	1.00
Social class II, n=985	83	1.05 (0.69 to 1.61)	1.03 (0.67 to 1.57)	0.89 (0.58 to 1.37)	0.76 (0.48 to 1.19)
Social class IIIN, n=381	31	1.08 (0.65 to 1.79)	1.01 (0.61 to 1.69)	0.90 (0.54 to 1.50)	0.82 (0.48 to 1.41)
Social class IIIM, n=1439	166	1.60 (1.08 to 2.37)	1.43 (0.90 to 2.15)	1.16 (0.77 to 1.75)	0.99 (0.64 to 1.54)
Social class IV, n=336	30	1.32 (0.79 to 2.20)	1.15 (0.68 to 1.95)	0.93 (0.54 to 1.57)	0.73 (0.41 to 1.30)
Social class V (most deprived), n=106	18	2.45 (1.36 to 4.42)	2.12 (1.16 to 3.90)	1.47 (0.79 to 2.73)	1.07 (0.55 to 2.09)
P value for trend		0.0001	0.007	0.1245	0.4755
HR per unit change in social class		1.17 (1.08 to 1.27)	1.13 (1.03 to 1.23)	1.07 (0.98 to 1.17)	1.04 (0.94 to 1.14)
Non-manual vs manual class					
Non-manual, n=1720	143	1.00	1.00	1.00	1.00
Manual, n=1881	214	1.53 (1.23 to 1.89)	1.39 (1.11 to 1.74)	1.25 (0.99 to 1.57)	1.18 (0.92 to 1.52)

Model 1: adjusted for age.

Model 2: further adjusted for quintile of neighborhood deprivation.

Model 3: further adjusted for BMI and family history of diabetes.

Model 4: further adjusted for smoking, alcohol, activity level, SBP, and cholesterol.

BMI, body mass index; BRHS, British Regional Heart Study; SBP, systolic blood pressure.

**Table 4:** HR (95% CI) for incident type 2 diabetes in the BRHS cohort according to independent individual-level socioeconomic factors and all factors combined in a cumulative score for adverse socioeconomic factors

		HR (95% CI)			
	**Cases of incident diabetes**	**Model 1**	**Model 2**	**Model 3**	**Model 4**
Age at leaving full-time education					
>14 years	218	1.00	1.00	1.00	1.00
≤14 years	97	1.10 (0.84 to 1.45)	1.01 (0.76 to 1.34)	0.95 (0.72 to 1.27)	0.89 (0.66 to 1.20)
Car ownership					
Yes	306	1.00	1.00	1.00	1.00
No	56	1.29 (0.97 to 1.72)	1.15 (0.86 to 1.55)	1.07 (0.79 to 1.44)	1.09 (0.79 to 1.50)
Home ownership					
Yes	312	1.00	1.00	1.00	1.00
No	48	1.43 (1.05 to 1.94)	1.25 (0.91 to 1.72)	1.13 (0.82 to 1.56)	1.09 (0.78 to 1.54)
Receive only state pension					
No	236	1.00	1.00	1.00	1.00
Yes	132	1.00 (0.80 to 1.24)	0.94 (0.75 to 1.17)	0.93 (0.74 to 1.16)	0.90 (0.71 to 1.14)
Central heating at home					
Yes	326	1.00	1.00	1.00	1.00
No	27	1.35 (0.91 to 1.99)	1.21 (0.82 to 1.80)	1.32 (0.88 to 1.98)	1.08 (0.68 to 1.71)
Cumulative (additive) score for adverse socioeconomic factors					
<3	304	1.00	1.00	1.00	1.00
≥3	64	1.27 (0.96 to 1.68)	1.12 (0.83 to 1.49)	1.08 (0.81 to 1.45)	0.99 (0.73 to 1.36)
P value for trend HR for one-point increase in score		0.0019 1.14 (1.05 to 1.24)	0.0718 1.09 (0.99 to 1.19)	0.2881 1.05 (0.96 to 1.16)	0.8116 1.01 (0.91 to 1.12)

Model 1: adjusted for age.

Model 2: further adjusted for quintile of neighborhood deprivation.

Model 3: further adjusted for BMI and family history of diabetes.

Model 4: further adjusted for smoking, alcohol, activity level, SBP, and cholesterol.

BMI, body mass index; BRHS, British Regional Heart Study; SBP, systolic blood pressure.

Table 3 shows the HR for developing diabetes according to individual social class, where the “hazards” or risks of diabetes in social classes II–V are compared with the highest social class I (reference group). The risks of diabetes increased from social class I to V (p for trend=0.0001; age-adjusted HR for social class V=2.45, 95% CI 1.36 to 4.42). Adjustment for IMD quintiles of neighborhood-level deprivation weakened the HR for social class V, although it remained significant. Further adjustment for BMI and family history of diabetes in a first-degree relative attenuated the HR estimate, which was no longer statistically significant.

Table 4 displays the age-adjusted HR (95% CI) for incident diabetes according to individual socioeconomic factors and all factors combined to assess their additive effect in the form of a *cumulative adverse socioeconomic factors score.* Each factor was first analyzed independently, and the increased risk was only significant for home ownership; the HR for those who did not own their own home compared with those who did was 1.43 (95% CI 1.05 to 1.94). Further adjustment for the IMD quintile for neighborhood deprivation attenuated the HR. When all factors were combined, there was a graded increase in the HR for developing diabetes as the cumulative adverse socioeconomic score increased (HR for a one-point increase in the cumulative adverse socioeconomic score=1.14, 95% CI 1.05 o 1.24). This was no longer significant after adjustment for IMD quintile.

## Discussion

This national longitudinal study with 18 years of follow-up in older age British men showed that the risk of incident T2DM varied according to neighborhood-level socioeconomic deprivation, social class, and cumulative adverse socioeconomic factors. Those living in more deprived neighborhoods and of the lowest social classes had approximately 1.5-fold to 2-fold increased risk of T2DM, clearly demonstrating the extent of socioeconomic inequalities in diabetes within this older age UK population. The increased risk associated with neighborhood-level deprivation (IMD) was attenuated on adjusting for individual-level social class and became statistically non-significant when adjusting for known behavioral and biological risk factors for T2DM. This suggests that the increased risk of T2DM seen in the most deprived areas is at least partly due to individual-level socioeconomic factors and the unequal distribution of the established risk factors for T2DM, including BMI.

### Strengths and limitations

This paper highlights that socioeconomic inequalities in the risks of developing T2DM persist into older age and that this is evident whether the inequalities are looked at through the lens of neighborhood-level deprivation or individual socioeconomic factors. To the best of our knowledge, this is the first report to use national prospective data to examine the relationship between neighborhood-level IMD and incident T2DM in older British men.

The results are based on a socially representative group of older British men. The prospective nature of the investigations allows for assessment of the temporal relationship between neighborhood-level deprivation and the development of T2DM. Follow-up through medical record review is particularly useful in older cohorts to enable more complete follow-up and information on participants who may otherwise be too frail or unwell to participate. The retention and follow-up of participants in the cohort were high; at 20 years’ follow-up of the BRHS cohort, the estimated study retention was over 99% of the participants.^31^ A robust approach to exclude participants with prevalent diabetes at baseline was used, which included fasting glucose or HbA1c at baseline. This helped identify asymptomatic or undiagnosed cases of prevalent diabetes at baseline.

The generalizability of the study is limited by the cohort’s composition of white European men. Statistical power overall, but particularly in the most deprived groups, may have been limited by the small number of surviving participants, limiting the power to detect modest associations in this study. Covariates adjusted in the analyses were from the baseline time point and were not time-varying, which could lead to a potential bias in estimating the true effects of covariates. Also, the role of factors such as food intake was not taken into account. Since the deprivation scores (IMD scores) used were from baseline, it is possible that movement of participants into more (or less) deprived areas was not captured. However, the BRHS sample is a relatively stable sample, with very limited (<5%) mobility over the period of follow-up. Previous publications using BRHS data have shown that, as early as 5 years after the study began, there were significant differences in the rates of all-cause mortality according to the SEP of participants, which risks introducing survivor bias.^33^ Moreover, the cohort that attended the physical examination at the baseline time point in this paper (77%) were healthier and of higher social classes, compared with those who did not attend—detailed information regarding how this cohort compared with the full original cohort, including non-responders, has previously been published by the BRHS.^34^ Therefore, it is likely that the most deprived individuals with higher risk of disease are under-represented in the present analysis.

### Comparison with other studies

This study provides further information about the nature of the association between neighborhood-level deprivation and risk of T2DM in older populations. There are two previous studies that we are aware of which focused on older populations.^11 18^ These studies differed from ours in terms of gender and ethnicity of the samples—one included women (only) aged 60–79 years from the British Women’s Heart and Health Study,^11^ and the other study was the Sacramento Area Latino Study on Aging^18^ in California. These studies both demonstrated an independent association between neighborhood-level deprivation and T2DM, which was robust to adjustment for individual-level socioeconomic status *and* behavioral and biological factors. Similar findings have also been demonstrated in studies comprising younger adult populations which adjusted for multiple individual-level factors including a measure of individual-level SEP.^13 17 21^ An Australian population study of adults >18 years found no independent association between area-level deprivation measures and diabetes when adjusting for individual-level SEP.^20^ Our study demonstrated an association between neighborhood-level deprivation and T2DM, which was robust to adjustment for individual-level SEP, but not further behavioral and biological risk factors. There is significant heterogeneity in the studies examining this association, in terms of study populations, neighborhood-level definitions, measures of individual SEP, and methodology, which may explain at least in part the differences observed in study findings. Our study did not find a significant impact of family history of T2DM on the association of neighborhood-level deprivation with diabetes; to our knowledge, ours is the only study to have taken account of this important risk factor for diabetes.

### Implications and conclusions

The findings of this study highlight the fact that inequalities in T2DM risk both in relation to neighborhood-level deprivation and individual SEP persist in older age UK populations. T2DM is a potentially *preventable* (and even *reversible*) disease that is associated with modifiable individual-level, health-related behavioral and biological factors. This study has shown that inequalities in both the distribution of T2DM and the rates of modifiable risk factors for T2DM persist into later life in this older age UK cohort. Among other factors, BMI remains a potentially important modifiable factor underlying the association between deprivation and incident T2DM. Other modifiable and individual-level factors such as food intake or diet, as well as aspects such as social connections or networks in older age, could potentially be relevant in older age and merit further research. These unequally distributed risk factors for T2DM should remain important areas for consideration when considering the potential for disease prevention in later life.

Health inequalities within the UK are widespread, growing, and challenging to tackle.^35^ Initiatives targeted at whole populations may inadvertently widen health inequalities, as people in the most deprived populations are the least likely to respond to a non-targeted approach (eg, breast cancer screening uptake in London^36^). This means that the already healthy sector of the population gets healthier, while the rest of the population are left behind. Instead, we advocate for “proportionate universalism,” where the scale and intensity of the intervention are proportionate to the level of disadvantage faced by the individual.^37^ In practice, this would mean the resourcing and targeting of public health interventions for T2DM at the whole population, but accepting that the most deprived groups will need to be allocated *more* resources to achieve equal outcomes. Physicians should ensure that current interventions, such as access to social prescribing or weight loss management, are equitable within their populations and that the deprived older age population is specifically resourced and targeted.

Levels of potentially modifiable individual-level factors have also been shown to be influenced by neighborhood-level factors^38^; the Lancet Commission on Diabetes acknowledges that “The diverse environmental, behavioural, and socioeconomic causes of type 2 diabetes require a multitiered societal and population-based prevention strategy.”^39^ Further research is also required to identify if there are potentially modifiable neighborhood characteristics or environmental factors within the UK which are contributing to the distribution of these individual-level risk factors. These studies would require detailed local descriptors of the environment and might include factors such as the food environment, access to green spaces or exercise facilities, rural–urban classification, air pollution, perceived risk of street crime, and community attitudes to smoking and exercise. Public health and planning teams need to continue to consider what a healthy environment looks like for an older person in their population as needs are likely to significantly differ across the life course.

## Data Availability

Data are available from the British Regional Heart Study subject to request approval: https://www.ucl.ac.uk/epidemiology-health-care/research/primary-care-and-population-health/research/ageing/british-regional-heart-study-brhs/brhs-2.
