# Supplementary material for: Influence of neighborhood-level socioeconomic deprivation and individual socioeconomic position on risk of developing type 2 diabetes in older men: a longitudinal analysis in the British Regional Heart Study cohort
Source: BMJ Open Diabetes Res Care. 2023 Oct 31;11(5):e003559. doi: 10.1136/bmjdrc-2023-003559 (PMC10619023; doi:10.1136/bmjdrc-2023-003559)
Supplement: Supplementary data [file bmjdrc-2023-003559supp001.pdf]

Appendix: Full Models for Tables 2-4 in main text

Table A1 Full Model: Hazard Ratios for incident Type 2 Diabetes in the BRHS Cohort According to Index of Multiple Deprivation (IMD) Quintiles of Neighbourhood-Level Deprivation scores

	Hazard Ratio (95% Confidence Intervals)			
	(Model 1) Adjusted for age	(Model 2) Further adjusted for Individual social class	(Model 3) Further adjusted for BMI & Family History of diabetes.	(Model 4) Further adjusted for smoking, alcohol, activity levels, Systolic BP & Cholesterol
IMD Quintile 1 (Least Deprived)	1.00	1.00	1.00	1.00
IMD Quintile 2	1.07 (0.78-1.47)	0.97 (0.70-1.34)	0.95 (0.69-1.32)	0.90 (0.63-1.28)
IMD Quintile 3	1.33 (0.96-1.84)	1.15 (0.82-1.61)	1.13 (0.81-1.59)	1.19 (0.83-1.71)
IMD Quintile 4	1.80 (1.31-2.47)	1.52 (1.09-2.13)	1.42 (1.01-1.99)	1.31 (0.90-1.90)
IMD Quintile 5 (Most Deprived)	1.46 (1.04-2.05)	1.21 (0.84-1.73)	1.16 (0.81-1.67)	1.16 (0.78-1.73)
Age (HR per unit increase in 1 year of age)	0.98 0.97-1.00)	0.99 (0.97-1.01)	1.00 (0.98-1.02)	0.99 (0.97-1.02)
Social Class				
I		1.00	1.00	1.00
II		1.02 (0.67-1.57)	0.89 (0.58-1.37)	0.76 (0.48-1.41)
IIIN		1.01 (0.61-1.69)	0.90 (0.54-1.50)	0.82 (0.48-1.41)
IIIM		1.43 (0.95-2.15)	1.16 (0.77-1.75)	0.99 (0.64-1.54)
IV		1.15 (0.68-1.95)	0.93 (0.54-1.57)	0.73 (0.41-1.30)
V		2.12 (1.16 -3.90)	1.47 (0.79 -2.73)	1.07 (0.41-1.30)
BMI (HR per unit increase in BMI)			1.17 (1.14-1.20)	1.17 (1.13-1.20)
Family History of diabetes				
No			1.00	1.00
Yes			1.40 (1.02-1.89)	1.38 (0.99-1.94)
Smoking Status				
Never				1.00
Ex-smoker >15yrs				1.28 (0.96- 1.69)
Recent Ex-smoker <15 years				1.46 (1.02- 2.10)
Current smoker				1.39 (0.92- 2.08)
Alcohol				
None				1.00
Occasional(<1 drink/week)				0.79 (0.54- 1.16)
Light(1-15/week)				0.76 (0.53- 1.10)
Moderate(16-42/weeks)				0.47 (0.30- 0.75)
Heavy(>42/week)				0.32 (0.13- 0.82)
Physical Activity				
Inactive				1.00
Occasional				0.95 (0.65- 1.38)
Light				0.93 (0.63- 1.37)
Moderate				0.84 (0.55- 1.28)
Moderate vigorous				0.70 (0.45- 1.08)
Vigorous				0.89 (0.58- 1.37)
Systolic BP (HR per unit increase in systolic BP)				1.01 (1.01- 1.01)
Cholesterol (HR per unit increase in cholesterol)				1.08 (0.97- 1.20)

Table A2 Full Model: Hazard Ratios for incident Type 2 diabetes in the BRHS Cohort According to Socioeconomic Position (Occupational Social Class, 6 Levels)

	Hazard Ratio (95% Hazard Ratio Confidence Limits)			
	(Model 1)<br>Adjusted for age	(Model 2)<br>Further adjusted for Quintile of Neighbourhood Deprivation	(Model 3)<br>Further adjusted for BMI & Family History of diabetes.	(Model 4)<br>Further adjusted for smoking,<br>alcohol, activity levels, Systolic BP & Cholesterol
Social Class I<br>(Least Deprived)	1.00	1.00	1.00	1.00
Social Class II	1.05 (0.69-1.61)	1.03 (0.67-1.57)	0.89 (0.58-1.37)	0.76 (0.48-1.19)
Social Class IIIN	1.08 (0.65-1.79)	1.01 (0.61-1.69)	0.90 (0.54-1.50)	0.82 (0.48-1.41)
Social Class IIIM	1.60 (1.08-2.37)	1.43 (0.90-2.15)	1.16 (0.77-1.75)	0.99 (0.64-1.54)
Social Class IV	1.32 (0.79-2.20)	1.15 (0.68-1.95)	0.93 (0.54-1.57)	0.73 (0.41-1.30)
Social Class V<br>(Most Deprived)	2.45 (1.36-4.42)	2.12 (1.16-3.90)	1.47~~2~~ (0.79-2.73)	1.07 (0.550-2.09)
Age (HR per unit increase in 1 year of age)	0.99 (0.97-1.01)	0.99 (0.97-1.01)	1.00 (0.98- 1.02)	0.99 (0.97- 1.02)
Neighbourhood IMD Quintile				
Quintile 1		1.00	1.00	1.00
Quintile 2		0.97 (0.70- 1.34)	0.95 (0.69- 1.32)	0.90 (0.63- 1.28)
Quintile 3		1.15 (0.82- 1.61)	1.13 (0.81- 1.59)	1.19 (0.83- 1.71)
Quintile 4		1.52 (1.09- 2.13)	1.42 (1.01- 1.99)	1.31 (0.90- 1.90)
Quintile 5		1.21 (0.84- 1.73)	1.16 (0.81- 1.67)	1.16 (0.78- 1.73)
BMI<br>(HR per unit increase in BMI)			1.17 (1.14- 1.20)	1.17 (1.13- 1.20)
Family History of diabetes				
No			1.00	1.00
Yes			1.39 (1.02- 1.89)	1.38 (0.99- 1.94)
Smoking Status				
Never				1.00
Ex-smoker >15yrs				1.28 (0.96- 1.69)
Recent Ex-smoker <15 years				1.46 (1.02- 2.10)
Current smoker				1.39 (0.92- 2.08)
Alcohol				
None				1.00
Occasional(<1 drink/week)				0.79 (0.54- 1.16)
Light(1-15/week)				0.76 (0.53- 1.10)
Moderate(16-42/weeks)				0.47 (0.30- 0.75)
Heavy(>42/week)				0.32 (0.13- 0.82)
Physical Activity				
Inactive				1.00
Occasional				0.95 (0.65- 1.38)
Light				0.93 (0.63- 1.37)
Moderate				0.84 (0.55- 1.28)
Moderate vigorous				0.70 (0.45- 1.08)
Vigorous				0.89 (0.58- 1.37)
Systolic BP (HR per unit increase in systolic BP)				1.01 (1.00- 1.01)
Cholesterol (HR per unit increase in cholesterol)				1.08 (0.97- 1.20)

Table A3 Full Model: Hazard Ratios for incident Type 2 diabetes in the BRHS Cohort According to Socioeconomic Position (Occupational Social Class, Non Manuel vs Manual Occupation)

	Hazard Ratio (95% Hazard Ratio Confidence Limits)			
	(Model 1)<br>Adjusted for age	(Model 2)<br>Further adjusted for Quintile of Neighbourhood Deprivation	(Model 3)<br>Further adjusted for BMI<br>& Family History of diabetes.	(Model 4)<br>Further adjusted for smoking,<br>alcohol, activity levels,<br>Systolic BP &<br>Cholesterol
Non-Manual Occupation	1.00	1.00	1.00	1.00
Manual Occupation	1.53 (1.23-1.89)	1.39 (1.11-1.74)	1.25 (0.99-1.57)	1.18 (0.92-1.52)
Age (HR per unit increase in 1 year of age)	0.99 (0.97-1.01)	0.99 (0.97-1.01)	1.00 (0.98-1.02)	0.99 (0.98-1.02)
Neighbourhood IMD Quintile				
Quintile 1 (Ref group)		1.00	1.00	1.00
Quintile 2		0.97 (0.70- 1.35)	0.95 (0.68- 1.32)	0.89 (0.62- 1.26)
Quintile 3		1.14 (0.82- 1.59)	1.11 (0.80- 1.56)	1.16 (0.81- 1.66)
Quintile 4		1.52 (1.09- 2.12)	1.39 (0.99- 1.95)	1.27 (0.88- 1.83)
Quintile 5		1.23 (0.86- 1.75)	1.17 (0.82- 1.67)	1.14 (0.78- 1.69)
BMI				
(HR per unit increase in BMI)			1.17 (1.14- 1.20)	1.16 (1.13- 1.20)
Family History of diabetes in a first degree relative			1.00	1.00
No			1.39 (1.02- 1.89)	1.41 (1.01- 1.96)
Yes				
Smoking Status				
Never smoker				1.00
Ex-smoker >15yrs				1.29 (0.97- 1.70)
Recent Ex-smoker <15 years				1.47 (1.03- 2.10)
Current smoker				1.37 (0.92- 2.05)
Alcohol				
None				1.00
Occasional(<1 drink/week)				0.80 (0.54- 1.17)
Light(1-15/week)				0.78 (0.54- 1.12)
Moderate(16-42/weeks)				0.49 (0.31- 0.78)
Heavy(>42/week)				0.33 (0.13- 0.85)
Physical Activity				
Inactive				1.00
Occasional				0.93 (0.64- 1.35)
Light				0.92 (0.62- 1.35)
Moderate				0.83 (0.55- 1.26)
Moderate vigorous				0.69 (0.44- 1.07)
Vigorous				0.88 (0.58- 1.35)
Systolic BP (HR per unit increase in systolic BP)				1.01 (1.00- 1.01)
Cholesterol (HR per unit increase in cholesterol)				1.08 (0.97- 1.20)

Table A4 Full Model: Hazard Ratios (95% Confidence Intervals) for Incident Type 2 Diabetes in the BRHS Cohort According to Independent Individual-level Socioeconomic Factors – Age leaving education

	Hazard Ratio (95% Confidence Interval)			
	(Model 1) Adjusted for age	(Model 2) Further adjusted for Quintile of Neighbourhood Deprivation	(Model 3) Further adjusted for BMI & Family History of diabetes.	(Model 4) Further adjusted for smoking, alcohol, activity levels, Systolic BP & Cholesterol
Age leaving education				
>14 years	1.00	1.00	1.00	1.00
<14 years	1.10 (0.84-1.45)	1.01 (0.76-1.34)	0.95 (0.717-1.27)	0.89 (0.66-1.20)
Age (HR per unit increase in 1 year of age)	0.99 (0.96-1.01)	0.99 (0.96-1.01)	1.01 (0.98-1.03)	1.00 (0.97-1.03)
Neighbourhood IMD Quintile				
Quintile 1 (Ref group)		1.00	1.00	1.00
Quintile 2		1.00 (0.72- 1.40)	0.96 (0.69- 1.35)	0.93 (0.64- 1.34)
Quintile 3		1.19 (0.84- 1.69)	1.15 (0.81- 1.63)	1.19 (0.82- 1.73)
Quintile 4		1.69 (1.20- 2.38)	1.47 (1.04- 2.09)	1.33 (0.90- 1.95)
Quintile 5		1.46 (1.01- 2.11)	1.35 (0.93- 1.95)	1.31 (0.88- 1.95)
BMI (HR per unit increase in BMI)			1.17 (1.14- 1.19)	1.16 (1.13- 1.20)
Family History of diabetes in a first degree relative				
No			1.00	1.00
Yes			1.54 (1.12- 2.13)	1.56 (1.10- 2.21)
Smoking Status				
Never smoker				1.00
Ex-smoker >15yrs				1.31 (0.98- 1.76)
Recent Ex-smoker <15 years				1.53 (1.05- 2.23)
Current smoker				1.48 (0.96- 2.30)
Alcohol				
None				1.00
Occasional(<1 drink/week)				0.81 (0.54- 1.22)
Light(1-15/week)				0.70 (0.48- 1.03)
Moderate(16-42/weeks)				0.49 (0.30- 0.79)
Heavy(>42/week)				0.19 (0.06- 0.63)
Physical Activity				
Inactive				1.00
Occasional				1.23 (0.80- 1.89)
Light				1.15 (0.74- 1.80)
Moderate				0.98 (0.61- 1.58)
Moderate vigorous				0.83 (0.51- 1.36)
Vigorous				1.08 (0.67- 1.74)
Systolic BP (HR per unit increase in systolic BP)				1.01 (1.00- 1.01)
Cholesterol (HR per unit increase in cholesterol)				1.08 (0.97- 1.21)

Table A5 Full Model: Hazard Ratios (95% Confidence Intervals) for Incident Type 2 Diabetes in the BRHS Cohort According to Independent Individual-level Socioeconomic Factors – Car Ownership

	Hazard Ratio (95% Confidence Interval) (Model 1) Adjusted for age	(Model 2) Further adjusted for Quintile of Neighbourhood Deprivation	(Model 3) Further adjusted for BMI & Family History of diabetes.	(Model 4) Further adjusted for smoking, alcohol, activity levels, Systolic BP & Cholesterol
Car ownership				
Yes	1.00	1.00	1.00	1.00
No	1.29 (0.97-1.72)	1.15 (0.86-1.55)	1.07 (0.79-1.44)	1.09 (0.79-1.50)
Age(HR per unit increase in 1 year of age)	0.98 (0.96-1.00)	0.98 (0.96-1.00)	1.00 (0.98-1.02)	0.97 (0.97-1.01)
Neighbourhood IMD Quintile				
Quintile 1 (Ref group)		1.00	1.00	1.00
Quintile 2		1.05 (0.77- 1.45)	1.02 (0.74- 1.41)	0.96 (0.68- 1.36)
Quintile 3		1.28 (0.92- 1.77)	1.22 (0.88- 1.70)	1.29 (0.91- 1.83)
Quintile 4		1.71 (1.24- 2.36)	1.50 (1.08- 2.07)	1.34 (0.94- 1.92)
Quintile 5		1.42 (1.00- 2.01)	1.32 (0.93- 1.88)	1.27 (0.87- 1.85)
BMI (HR per unit increase in BMI)			1.17 (1.14- 1.20)	1.16 (1.13- 1.19)
Family History of diabetes in a first degree relative				
No			1.00	1.00
Yes			1.47 (1.08- 1.98)	1.48 (1.07- 2.05)
Smoking Status				
Never smoker				1.00
Ex-smoker >15yrs				1.26 (0.96- 1.65)
Recent Ex-smoker <15 years				1.47 (1.03- 2.08)
Current smoker				1.35 (0.91- 2.01)
Alcohol				
None				1.00
Occasional(<1 drink/week)				0.88 (0.60- 1.30)
Light(1-15/week)				0.80 (0.55- 1.15)
Moderate(16-42/weeks)				0.51 (0.32- 0.81)
Heavy(>42/week)				0.32 (0.13- 0.83)
Physical Activity				
Inactive				1.00
Occasional				0.90 (0.62- 1.31)
Light				0.89 (0.60- 1.30)
Moderate				0.80 (0.53- 1.21)
Moderate vigorous				0.70 (0.46- 1.07)
Vigorous				0.88 (0.58- 1.34)
Systolic BP (HR per unit increase in systolic BP)				1.01 (1.00- 1.01)
Cholesterol (HR per unit increase in cholesterol)				1.09 (0.98- 1.20)

Table A6 Full Model: Hazard Ratios (95% Confidence Intervals) for Incident Type 2 Diabetes in the BRHS Cohort According to Independent Individual-level Socioeconomic Factors – Home Ownership

	Hazard Ratio (95% Confidence Interval) (Model 1) Adjusted for age	(Model 2) Further adjusted for Quintile of Neighbourhood Deprivation	(Model 3) Further adjusted for BMI & Family History of diabetes.	(Model 4) Further adjusted for smoking, alcohol, activity levels, Systolic BP & Cholesterol
Home ownership				
Yes	1.00	1.00	1.00	1.00
No	1.43 (1.05-1.94)	1.25 (0.91-1.72)	1.13 (0.82-1.56)	1.09 (0.78-1.54)
Age (HR per unit increase in 1 year of age)	0.99 (0.97-1.01)	0.98 (0.96-1.00)	1.00 (0.98- 1.02)	0.99 (0.97-1.01)
Neighbourhood IMD Quintile				
Quintile 1 (Ref group)		1.00	1.00	1.00
Quintile 2		1.07 (0.78- 1.47)	1.02 (0.74- 1.42)	0.97 (0.68- 1.38)
Quintile 3		1.31 (0.95- 1.82)	1.26 (0.90- 1.75)	1.34 (0.94- 1.90)
Quintile 4		1.73 (1.25- 2.40)	1.53 (1.10- 2.13)	1.40 (0.98- 2.01)
Quintile 5		1.37 (0.96- 1.95)	1.30 (0.91- 1.85)	1.27 (0.86- 1.86)
BMI (HR per unit increase in BMI)			1.16 (1.14- 1.19)	1.16 (1.13- 1.19)
Family History of diabetes in a first degree relative				
No			1.00	1.00
Yes			1.43 (1.05- 1.94)	1.46 (1.05- 2.03)
Smoking Status				
Never smoker				1.00
Ex-smoker >15yrs				1.25 (0.95- 1.64)
Recent Ex-smoker <15 years				1.46 (1.03- 2.07)
Current smoker				1.32 (0.88- 1.99)
Alcohol				
None				1.00
Occasional(<1 drink/week)				0.86 (0.59- 1.26)
Light(1-15/week)				0.78 (0.54- 1.12)
Moderate(16-42/weeks)				0.50 (0.32- 0.79)
Heavy(>42/week)				0.31 (0.12- 0.78)
Physical Activity				
Inactive				1.00
Occasional				0.90 (0.62- 1.31)
Light				0.90 (0.61- 1.32)
Moderate				0.81 (0.53- 1.22)
Moderate vigorous				0.71 (0.46- 1.09)
Vigorous				0.89 (0.59- 1.36)
Systolic BP (HR per unit increase in systolic BP)				1.01 (1.00- 1.01)
Cholesterol (HR per unit increase in cholesterol)				1.08 (0.97- 1.20)

Table A7 Full Model: Hazard Ratios (95% Confidence Intervals) for Incident Type 2 Diabetes in the BRHS Cohort According to Independent Individual-level Socioeconomic Factors – State Pension Only

	Hazard Ratio (95% Confidence Interval) (Model 1) Adjusted for age	(Model 2) Further adjusted for Quintile of Neighbourhood Deprivation	(Model 3) Further adjusted for BMI & Family History of diabetes.	(Model 4) Further adjusted for smoking, alcohol, activity levels, Systolic BP & Cholesterol
State Pension Only				
No	1.00	1.00	1.00	1.00
Yes	1.00 (0.80-1.24)	0.94 (0.75-1.17)	0.93 (0.74-1.16)	0.90 (0.71-1.14)
Age (HR per unit increase in 1 year of age)	0.99 (0.97-1.01)	0.99 (0.97-1.01)	1.00 (0.98-1.02)	0.99 (0.97-1.02)
Neighbourhood IMD Quintile				
Quintile 1 (Ref group)		1.00	1.00	1.00
Quintile 2		1.08 (0.78- 1.49)	1.04 (0.75- 1.44)	0.99 (0.70- 1.40)
Quintile 3		1.34 (0.97- 1.86)	1.27 (0.91- 1.76)	1.32 (0.94- 1.87)
Quintile 4		1.82 (1.32- 2.50)	1.59 (1.14- 2.18)	1.43 (1.00- 2.03)
Quintile 5		1.48 (1.05- 2.08)	1.35 (0.95- 1.90)	1.30 (0.90- 1.89)
BMI (HR per unit increase in BMI)			1.17 (1.14- 1.20)	1.16 (1.13- 1.20)
Family History of diabetes in a first degree relative				
No			1.00	1.00
Yes			1.42 (1.05- 1.92)	1.43 (1.03- 1.98)
Smoking Status				
Never smoker				1.00
Ex-smoker >15yrs				1.28 (0.98- 1.68)
Recent Ex-smoker <15 years				1.49 (1.05- 2.11)
Current smoker				1.36 (0.92- 2.02)
Alcohol				
None				1.00
Occasional(<1 drink/week)				0.87 (0.59- 1.27)
Light(1-15/week)				0.79 (0.55- 1.13)
Moderate(16-42/weeks)				0.50 (0.32- 0.79)
Heavy(>42/week)				0.30 (0.12- 0.77)
Physical Activity				
Inactive				1.00
Occasional				0.90 (0.62- 1.30)
Light				0.89 (0.61- 1.30)
Moderate				0.81 (0.53- 1.22)
Moderate vigorous				0.68 (0.44- 1.04)
Vigorous				0.87 (0.57- 1.32)
Systolic BP (HR per unit increase in systolic BP)				1.01 (1.00- 1.01)
Cholesterol (HR per unit increase in cholesterol)				1.09 (0.98- 1.21)

Table A8 Full Model: Hazard Ratios (95% Confidence Intervals) for Incident Type 2 Diabetes in the BRHS Cohort According to Independent Individual-level Socioeconomic Factors – Central Heating in the home

	Hazard Ratio (95% Confidence Interval) (Model 1) Adjusted for age	(Model 2) Further adjusted for Quintile of Neighbourhood Deprivation	(Model 3) Further adjusted for BMI & Family History of diabetes.	(Model 4) Further adjusted for smoking, alcohol, activity levels, Systolic BP & Cholesterol
Central Heating				
Yes	1.00	1.00	1.00	1.00
No	1.35 (0.91-1.99)	1.21 (0.82-1.80)	1.32 (0.88-1.98)	1.08 (0.68-1.71)
Age (HR per unit increase in 1 year of age)	0.98 (0.96-1.01)	0.98 (0.96-1.00)	1.00 (0.98-1.02)	0.99 (0.97-1.01)
Neighbourhood IMD Quintile				
Quintile 1 (Ref group)		1.00	1.00	1.00
Quintile 2		1.06 (0.77- 1.47)	1.03 (0.74- 1.43)	0.95 (0.67- 1.36)
Quintile 3		1.30 (0.93- 1.81)	1.24 (0.89- 1.74)	1.30 (0.91- 1.85)
Quintile 4		1.87 (1.36- 2.59)	1.63 (1.18- 2.27)	1.48 (1.03- 2.11)
Quintile 5		1.53 (1.08- 2.16)	1.38 (0.97- 1.96)	1.34 (0.92- 1.96)
BMI (HR per unit increase in BMI)			1.17 (1.14- 1.20)	1.17 (1.14- 1.20)
Family History of diabetes in a first degree relative				
No			1.00	1.00
Yes			1.43 (1.05- 1.95)	1.44 (1.03- 2.02)
Smoking Status				
Never smoker				1.00
Ex-smoker >15yrs				1.29 (0.98- 1.69)
Recent Ex-smoker <15 years				1.41 (0.99- 2.01)
Current smoker				1.36 (0.91- 2.04)
Alcohol				
None				1.00
Occasional(<1 drink/week)				0.85 (0.58- 1.26)
Light(1-15/week)				0.77 (0.54- 1.12)
Moderate(16-42/weeks)				0.47 (0.29- 0.75)
Heavy(>42/week)				0.33 (0.13- 0.85)
Physical Activity				
Inactive				1.00
Occasional				0.89 (0.61- 1.29)
Light				0.89 (0.60- 1.31)
Moderate				0.82 (0.54- 1.25)
Moderate vigorous				0.71 (0.46- 1.10)
Vigorous				0.89 (0.59- 1.36)
Systolic BP (HR per unit increase in systolic BP)				1.01 (1.00- 1.01)
Cholesterol (HR per unit increase in cholesterol)				1.08 (0.97- 1.20)
